# Morphological Phenotypes, Cell Division, and Gene Expression of *Escherichia coli* under High Concentration of Sodium Sulfate

**DOI:** 10.3390/microorganisms10020274

**Published:** 2022-01-25

**Authors:** Khanh Nguyen, Pradeep Kumar

**Affiliations:** Department of Physics, University of Arkansas, Fayetteville, AR 72701, USA; kmnguyen@uark.edu

**Keywords:** habitability of Europa, cellular response of *Escherichia coli* under hyperosmolar stress, sodium sulfate

## Abstract

Sodium and sulfate ions are among the suggested abundant ions on Europa, a moon of Jupiter. In order to investigate the potential habitability of Europa, we study the effects of sodium sulfate (Na_2_SO_4_) on a non-halophilic bacterium by subjecting *Escherichia coli* (*E. coli*) to a wide range of Na_2_SO_4_ concentrations (0–1.0 m). We discover that, as the concentration of sodium sulfate increases, the biomass doubling time increases and the cell growth is completely inhibited at 1.0 m Na_2_SO_4_. Furthermore, we find that *E. coli* exhibits three distinct morphological phenotypes—(i) shortened, (ii) normal, and (iii) elongated/filamented cells at 0.6 m and 0.8 m Na_2_SO_4_. We have examined the expression of different genes involved in sodium and sulfate transport (*nhaA, nhaB, cysZ, sbp*), osmotically driven transport of water (*aqpZ*), sulfate metabolism (*cysN*), fatty acid production (*fabA*), and a global transcriptional regulator (*osmZ*). Our results suggest that the expression of these genes is not affected significantly at high concentrations of sodium sulfate in the exponential growth phase. Using our experimental data and the existing data in the literature, we show that the osmotic pressure difference may play a major role in determining the growth inhibition of *E. coli* and *B. subtilis* at high concentrations of salt.

## 1. Introduction

Environmental conditions, such as high temperature, high pressure, hypersalinity and extremes of pH, are considered hostile to humans. However, there are other living organisms that are successfully able to grow and reproduce under these extreme conditions [1,2,3,4,5,6,7,8]. Analogs of environmental extremes on Earth also exist on other planetary bodies of the astrobiological importance. Europa, in particular, is a compelling candidate due to the presence of a global saline liquid water ocean in contact with a rocky core [9,10,11,12,13,14]. The discovery of dynamic surface features, including chaos regions, as well as remote detection of water vapor on Europa suggests that it is maintained over geologic timescales through tidal heating as Europa orbits around Jupiter [15,16]. The cycling of the ocean between the seafloor and the surface ice may yield an ocean rich with the elements and chemical energy needed to support life [17,18]. A combination of surface observation by Galileo’s near-infrared mapping spectrometer (NIMS) and geochemical modeling suggests a range of ionic composition of Europa’s ocean [19,20,21,22,23,24,25]. The subsurface ocean of Europa is hypothesized to be saline with the presumed abundance of Na${}^{+}$, SO${}_{4}^{2-}$, Mg${}^{2+}$, and Cl${}^{-}$. A geochemical model by Marion et al. places the Na${}^{+}$ and SO${}_{4}^{2-}$ concentrations in Europa’s ocean to be 1.6 M and 3.6 M, respectively [26]. Since Europa’s surface is relatively young and active, it is suggested that the surface chemical composition may be indicative of the composition of the subsurface ocean and the evidence for potential habitability of the ocean.

In order to investigate whether the life forms that we know would be able to survive under extreme conditions that may exist on Europa, several comparative genomics and metagenomics studies have been carried out [27,28,29,30,31]. While these studies do shed light on the differences in structure and stability of biomolecules leading to their adaptation to different extremes, they do not provide a detailed picture of cellular processes that are prone to failure and their limitations. Therefore, it is essential to investigate how different environmental conditions, their magnitude and duration, affect the cellular processes of a mesophilic (adapted to normal conditions) organism.

**Figure 1:**
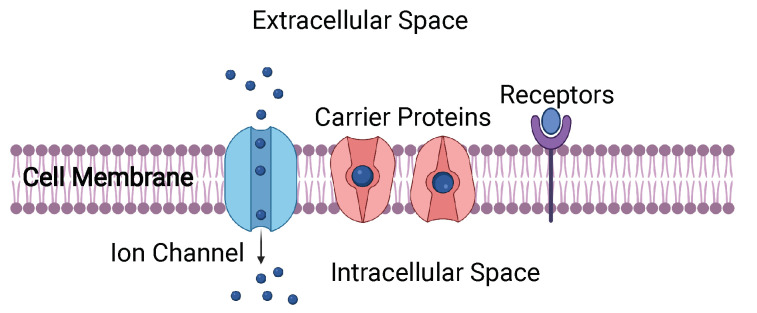
Schematic of the cell membrane and membrane proteins such as ion channels, carrier proteins, and receptors. Cells interact with the external environment via membrane proteins.

Some of the factors that determine the cellular response and adaptation to hypersaline environments include–ionic strength, chaotropic or kosmotropic nature of ions, and osmotic pressure. To understand the effect of high salt concentration on cells, one must consider the effect of high concentration of ions on different cellular building blocks, for example, biological membranes form the interface between the cells and their surroundings. Membrane proteins, such as ion channels, carrier proteins and cell signaling receptors, allow the cells to interact and respond to external environmental changes (see Figure 1). All cells require ions for cellular functions; ion channels allow the selective transport of ions to pass through the cell membrane. However, the presence of a large number of ions, whether they are inside or outside the cell, may affect the structure, stability and functionality of biomolecules, such as proteins, RNA, and DNA [32,33,34,35,36]. The chaotropic and kosmotropic nature of ions also dictate the stability and functionality of biomolecules [37,38]. Large concentrations of ions can create an osmotic gradient between inside and outside of the cell. An increase in external osmotic pressure causes water efflux [39,40] and may lead to plasmolysis [40,41]; on the other hand, a decrease in external osmotic pressure can lead to water influx and lysis. Cells respond to different external osmotic pressures by importing or exporting solutes, restricting the water fluxes. In extreme cases, some halophiles accumulate certain salts to molar concentrations, in which some proteins function only in high salinity environments.

Sodium chloride and/or potassium chloride have been considered as models of salt for studying the effect of salinity on bacteria [40,42,43,44,45,46,47]. Since the hypothesized ionic composition of Europa’s ocean includes sodium, magnesium and sulfate ions, it is important to investigate how these salts affect the behavior of bacterial cells, and the factors that set the upper limit on the growth and viability of cells. We have recently investigated the effect of high concentration of magnesium sulfate on a halotolerant bacterium, *E. coli* [48], but the effect of sodium sulfate (Na_2_SO_4_) on bacterial cells has remained unclear. We have performed experiments to examine the response of *E. coli* to different concentrations of sodium sulfate. The objectives of our study are the following—(i) to investigate the effect of high concentration of sodium sulfate on the cellular processes of a halotolerant bacterium; (ii) to determine if cells actively regulate gene expression to counteract the effect of hyperosmolarity in the presence of very high concentrations of sodium sulfate; and (iii) to determine the limiting factor, whether it is ionic strength, nature of ions, or osmotic pressure (or water activity) for growth inhibition.

## 2. Materials and Methods

### 2.1. Cell Culture and Media

Wild-type *Escherichia coli* K-12 strain MG1655 was obtained from the Coli Genetic Stock Center located at Yale University, USA. Cells were grown in solid LB-media agar (1.5% agar) (BD Difco, Franklin Lakes, NJ, USA) at 37 ${}^{\circ }$C for 16 hours. Cells were then picked from the petri dish and grown in liquid M9 media with the supplement of 2 mM MgSO_4_, 0.4% glucose and 0.4% succinate as carbon sources at 37 ${}^{\circ }$C in a shaking incubator until the optical density (OD${}_{600}$), reaches 0.8±0.1 (control sample). Then the bacterial sample was diluted to OD${}_{600}$≈0.10 in liquid M9 media containing different concentrations of Na_2_SO_4_ (0.2–1.0 m (mol/kg)) (Alfa Aesar, Haverhill, MA, USA). The pH of the media was measured using an Orion pH meter (Thermo Scientific, Carlsbad, CA, USA) by continuous stirring of media with a magnetic stirrer. The pH of the media decreases with increasing concentration of Na_2_SO_4_, and is 6.45±0.10 at 1.0 m of salt, the largest Na_2_SO_4_ concentration studied here. We have listed the pH value for different salt concentrations in Table 1. The media was filter-sterilized by passing through a 0.22 μm filter (Thermo Scientific, Carlsbad, CA, USA). The optical density of cells, except for growth measurements, was measured using Lambda Bio UV/VIS spectrometer (PerkinElmer, Waltham, MA, USA).

### 2.2. Experimental Setup to Measure the Growth Curve of Bacteria

**Figure 2:**
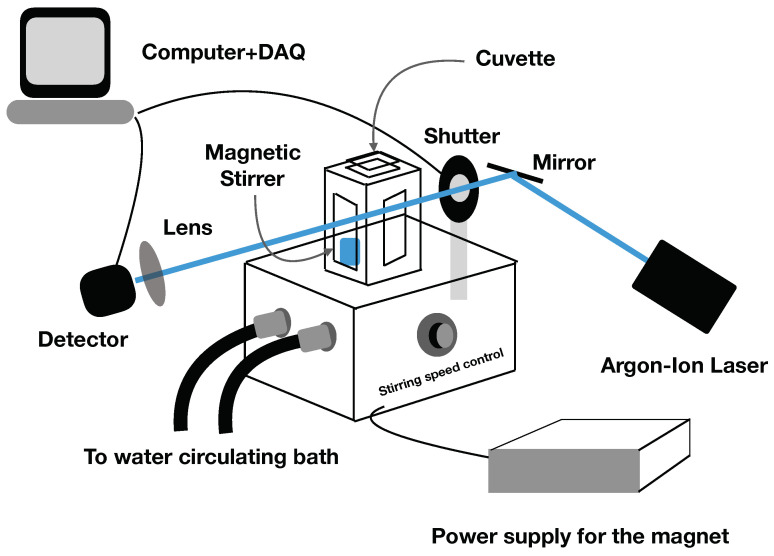
Schematic of the experimental setup used to measure the optical density, OD${}_{488}$, of bacteria in real time.

Schematic of the experimental setup used to measure the optical density, OD${}_{488}$, of bacteria in real time is shown in Figure 2. Light from an Argon-Ion laser (Spectra Physics, Santa Clara, CA, USA) of wavelength 488 nm is guided using a mirror into the sample chamber through a shutter. The shutter is controlled using a data acquisition device (DAQ) (National Instruments, Austin, TX, USA) and is only opened during the intensity measurement to avoid continuous exposure of bacterial cells to the laser. The sample chamber is equipped with a magnetic stirring system and the rate of stirring can be controlled using a control knob. Teflon coated magnetic stirrers for cuvettes were purchased from SP Bel-Art, PA, USA. A light intensity to frequency converter photodiode (AMS-TAOS, Plano, TX, USA) is used to measure the forward scattered light intensity using the DAQ. The light intensity thus measured is converted into optical density as described in the text. The temperature of the sample holder is controlled using a water circulating bath. Our experimental setup allows us to measure the optical density of the cell culture in real time without interruption.

Bacteria absorb and scatter light with an intensity that depends on the scattering angle and absorption coefficient [49]. The most common method for measuring bacterial concentration in a solution is the turbidity method in which the extinction of light is measured at a fixed angle, usually in the forward direction [50]. The optical density, OD${}_{488}$, is defined as:

(1) $${\mathrm{OD}}_{488}\left(t\right)=log_{10}\left(\frac{I_{0}}{I(t)}\right),$$

where $I_{0}$ is the reference intensity of light with just the media, and I(t) is the intensity of light through the sample at time *t*. For the exponential growth regime, one can write down,

(2) $${\mathrm{OD}}_{488}\left(t\right)={\mathrm{OD}}_{488}\left(0\right)2^{t/\tau_{d}},$$

where ${\mathrm{OD}}_{488}\left(0\right)$ is the optical density at time t=0, and $\tau_{d}$ is the biomass doubling time, the time scale over which the biomass doubles. Note that the OD${}_{488}$ is different from OD${}_{600}$ measured using 600 nm light. These two wavelengths give slightly different optical density values due to different absorption and scattering cross-sections. However, the biomass doubling time measured using any of these wavelengths should not be different. We verify this by measuring biomass doubling time using our setup as shown in Figure 2, and by measuring OD${}_{600}$ using the PerkinElmer UV/VIS spectrophotometer. We find that the biomass doubling time $\tau_{d}=45\pm 4$ min measured using our setup is comparable to 50±5 min measured using the spectrophotometer for cells grown in M9 media. The slight difference may arise due to the continuous regulation of temperature and continuous stirring of the sample, which are advantages of our experimental setup.

### 2.3. Imaging and the Image Analysis

For imaging, cells were grown in M9 media with the supplement of glucose, sodium succinate, 2 mM MgSO_4_, and different Na_2_SO_4_ concentrations to OD${}_{600}\approx 0.8$. Phase-contrast images of cells were acquired using a SPOT imaging camera mounted on a Nikon EFD-3 microscope with a 40× objective, immediately after the experiments to minimize the error in the morphology of the bacterial cells. For 1.0 m Na_2_SO_4_, at which we did not observe any growth, we obtained the images after growing the cells for 24 h. Images thus obtained were converted into binary images using ImageJ [51]. Binary images were subsequently analyzed using a custom MATLAB code to extract cell length.

### 2.4. Cell Death Assay

Bacterial samples were grown in M9 media with glucose (0.4%), sodium succinate (0.4%), 2 mM MgSO_4_ with different concentrations of sodium sulfate to OD${}_{600}\approx 0.8$. The samples were then washed twice with phosphate buffer saline (PBS) and were subsequently diluted in PBS with final OD${}_{600}$ ≈ 0.1. The samples were then stained with 100 ng/ml of propidium iodide (PI) and were incubated in the dark at room temperature for 45 min. Samples were further washed twice with PBS and were used to obtain the fluorescence intensities of cells (about 30,000 cells) using BD Ariafacs Fusion cell cytometer (BD Difco, Franklin Lakes, NJ, USA). The data were used to analyze the survival fraction over two biological replicates.

### 2.5. Primers and RT-qPCR

We also performed real-time quantitative PCR (RT-qPCR) to study the gene expression changes of *E. coli* at high sodium sulfate concentration. Specifically, we compared the gene expression of control cells (0 m Na_2_SO_4_) and cells at 0.8 m Na_2_SO_4_. The samples were first prepared by diluting the cells growing in M9 media (without sodium sulfate) at the mid-exponential phase (OD${}_{600}\approx 0.7$) to OD${}_{600}\approx 0.1$. Subsequently, the cells were grown to mid-exponential phase in M9 media with 0 m and 0.8 m sodium sulfate. RNA was extracted using RNeasy mini kit (Qiagen, Germantown, MD, USA). The total RNA sample was treated with DNAse (Thermo Fisher Scientific, Carlsbad, CA, USA) and was converted to complementary DNA (cDNA) using the iScript cDNA synthesis kit (Bio-Rad, Hercules, CA, USA). The cDNA template was amplified using iQ SYBR Green supermix (Bio-Rad, Hercules, CA, USA) and the respective primers. The list of primers used is given in Table 2. Primers were designed using the whole genome data from GeneBank using Primer Blast software (National Center for Biotechnology Information, Bethesda, MD, USA), and were purchased from Integrated DNA Technologies (Coralville, IA, USA). The amplicon length of each primer is 150 bp. Primers were tested using melt curve analysis, and in silico PCR [52]. The amplification was performed on the Quant Studio 3 real-time PCR system (Applied Biosystems, Thermo Fisher Scientific, Carlsbad, CA, USA). The data were normalized to 16s rRNA and the comparative gene expression (ΔΔCt) was calculated over two biological and two technical replicates.

## 3. Results

### 3.1. Cell Growth and Cell Death at Different Na_2_SO_4_ Concentration

**Figure 3:**
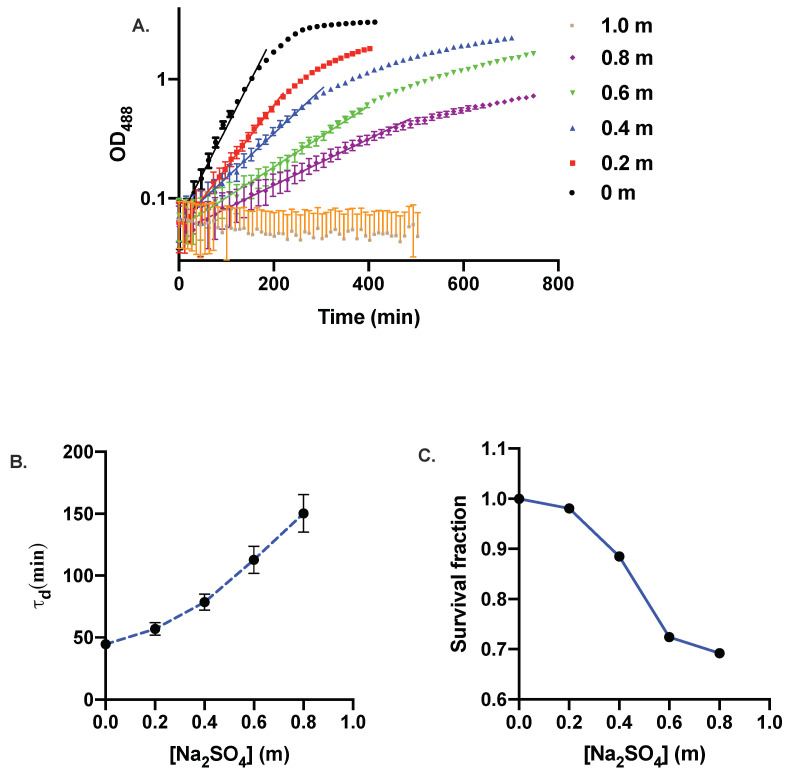
(**A**) Linear-log plot of the growth curves of *E. coli* at different concentrations of Na_2_SO_4_. (**B**) Biomass doubling time, $\tau_{d}$, of bacteria as a function of salt concentration. The biomass doubling time, $\tau_{d}$, is calculated by fitting exponential curves through the data points (shown as solid lines in **A**) in the exponential growth phase. (**C**) Survival fraction of bacteria as a function of Na_2_SO_4_ concentration.

*E. coli* cells were grown in M9 media at six different concentrations of Na_2_SO_4_: 0 m (control), 0.2 m, 0.4 m, 0.6 m, 0.8 m, and 1.0 m. All the experiments were carried out at 37 ${}^{\circ }$C. The optical density (OD${}_{488}$) of the cell culture was measured using our optical density measurement setup as shown in Figure 2. In Figure 3A, we show the experimental data of the optical density (OD${}_{488}$) (symbols) of bacterial cells as a function of time on a linear-log plot for six different concentrations of Na_2_SO_4_. We also include the instrumentation error on the optical density, $\Delta_{\mathrm{OD}}=0.028$. For 1.0 m Na_2_SO_4_, cells did not exhibit any growth as reflected in a nearly constant optical density over the timescale of our experiment. To further check whether *E. coli* grew if it is exposed to 1.0 m Na_2_SO_4_ for an extended period of time, we performed another experiment by inoculating the cells for 24 h, however we did not observe any growth. We show the exponential fits (solid lines), OD${}_{488}\left(t\right)$ = OD${}_{488}\left(0\right)2^{t/\tau_{d}}$, through the experimental data, where OD${}_{488}\left(0\right)$ is the initial optical density, and $\tau_{d}$ is the mass doubling time. Fitting was performed for the data lying in the exponential growth phase for all the salt concentrations. The saturation optical density decreases upon increasing concentration of Na_2_SO_4_. The biomass doubling time, $\tau_{d}$, as a function of salt concentration is shown in Figure 3B. The error on $\tau_{d}$ is estimated by assuming a Gaussian statistics for the instrumentation error, $\Delta_{\mathrm{OD}}$. Biomass doubling time increases with increasing concentration of sodium sulfate. Since the cells do not exhibit any growth at 1.0 m Na_2_SO_4_, we omit the biomass doubling time at this concentration. Our results are similar to previous studies on the effect of magnesium sulfate and sodium sulfate on the growth of *E. coli* [48,53]. We cannot make a direct comparison with the growth data of Refs. [53,54] because the growth medium used in our study is different. However, our results are qualitatively similar to those in Refs. [53,54].

In order to quantify the survival rate of *E. coli* under different sodium sulfate concentrations, we stained the bacterial cells with propidium iodide (PI) as described in the Materials and Methods section. Figure 3C shows the survival fraction of the bacterial cells as a function of Na_2_SO_4_ concentrations. Cell death increases with increasing salt concentration and it escalates quickly after 0.4 m Na_2_SO_4_. The results indicate that *E. coli* can withstand a moderately high concentration of sodium sulfate.

### 3.2. Cell Division, Cell Length, and Morphological Heterogeneity

**Figure 4:**
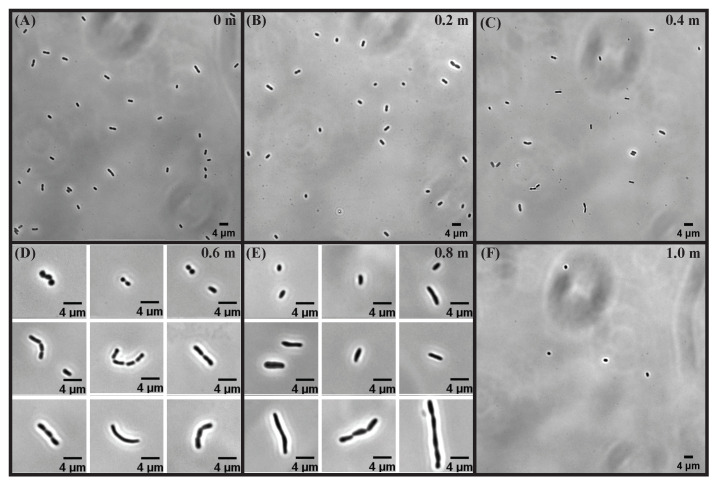
Representative images of cells at different concentrations (**A**) 0 m (control), (**B**) 0.2 m, (**C**) 0.4 m, (**D**) 0.6 m, (**E**) 0.8 m, and (**F**) 1.0 m of Na_2_SO_4_.

After looking at the viability of *E. coli*, we examine the morphology of bacterial cells at different concentrations of sodium sulfate. Figure 4 shows the representative images of bacterial cells grown at different Na_2_SO_4_ concentrations of 0 m (control), 0.2 m, 0.4 m, 0.6 m, 0.8 m, and 1.0 m. Since the cells do not grow at 1.0 m Na_2_SO_4_, images were obtained after inoculating the cells for 24 h.

For 0.6 m and 0.8 m Na_2_SO_4_, the cell population exhibits three different phenotypes—(i) cells smaller than the control cells (shortened cells); (ii) cells comparable in length to control cells; and (iii) cells longer than control cells (elongated cells). In the case of the elongated/filamented cells, the septum site is visible for a fraction of the population, but the cell division has not taken place. While for the rest of the population, we do not observe a clear indication of cell septum formation. These observations indicate that the bacterial cells have not divided and the unit cells are interconnected with each other. Therefore, we assume each of these cells to be a single cell (see Figure 4D,E).

**Figure 5:**
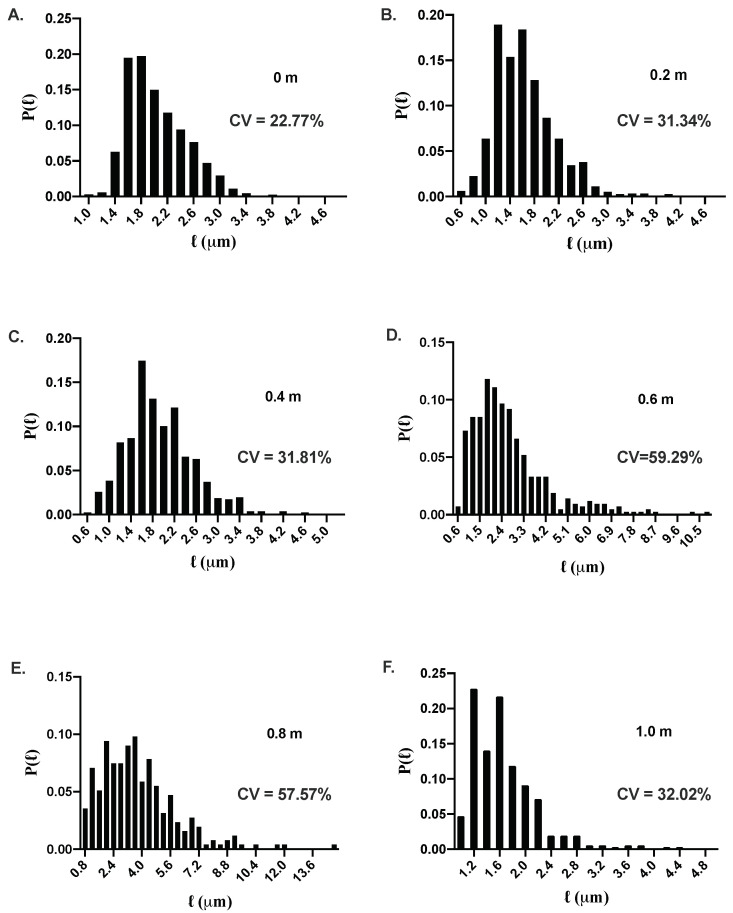
Probability distribution function, P(ℓ), of cell length, *ℓ*, for Na_2_SO_4_ concentrations: (**A**) 0 m, (**B**) 0.2 m, (**C**) 0.4 m, (**D**) 0.6 m, (**E**) 0.8 m, and (**F**) 1.0 m. The cell length distribution exhibits large heterogeneities at high concentrations of sodium sulfate. For 1.0 m Na_2_SO_4_, the bacterial cells do not exhibit any growth, and the cell length becomes smaller, presumably due to water efflux. Coefficient of variation (CV) of the probability distributions for all the salt concentrations are also shown.

**Figure 6:**
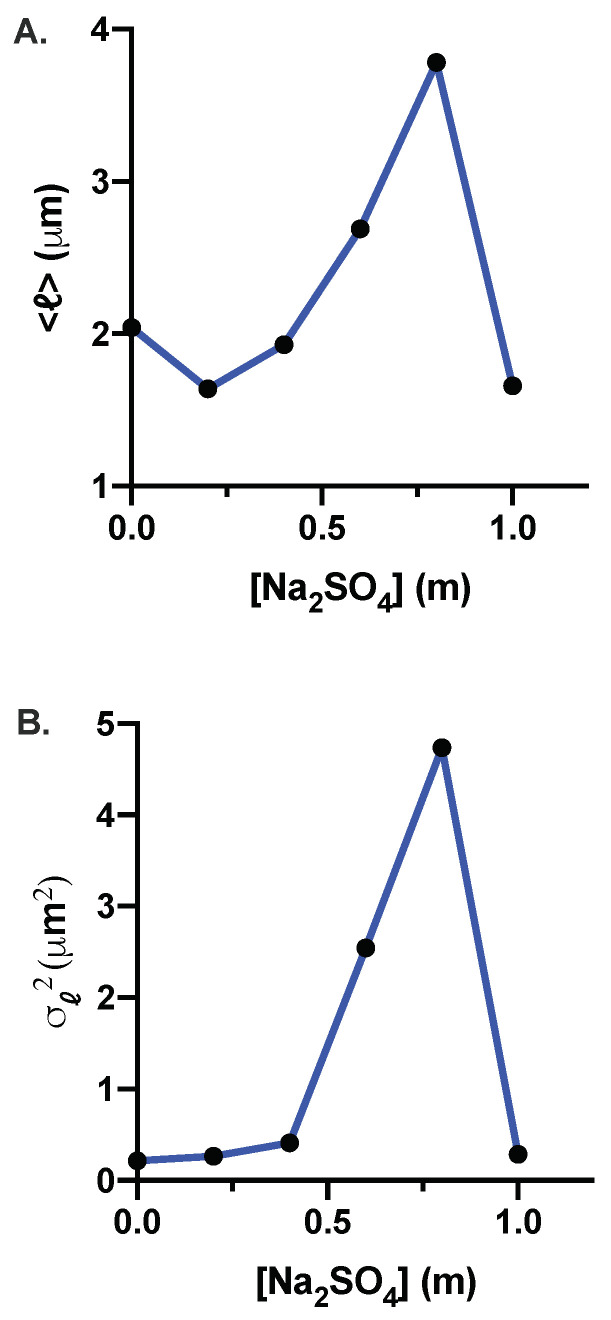
(**A**) Average cell length, $\langle \ell \rangle$, as a function of sodium sulfate concentration. $\langle \ell \rangle$ decreases slightly at 0.2 m and 0.4 m, then it increases monotonically between 0.4 m and 0.8 m, and becomes smaller at 1.0 m Na_2_SO_4_. (**B**) Variance of the cell length, $\sigma_{\ell }^{2}$, as a function of sodium sulfate concentration. The variance increases monotonically with Na_2_SO_4_ concentration up to 0.8 m.

The phase-contrast images obtained above were used to analyze and extract the cell length as described in the Materials and Methods section. In Figure 5, we show the probability distribution, P(ℓ), of cell length, *ℓ*, for 0 m (control), 0.2 m, 0.4 m, 0.6 m, 0.8 m, and 1.0 m Na_2_SO_4_. We summarize the statistics of cell length for different concentrations of Na_2_SO_4_ in Table 3. For 0.2 m and 0.4 m Na_2_SO_4_, the probability distribution is similar to the control cells but is shifted slightly to smaller values for the cell length. However, at 0.6 m and 0.8 m concentrations, the probability distribution is long-tailed due to the presence of elongated cells in the population. Figure 6A,B shows the mean length, $\langle \ell \rangle$, and the variance, $\sigma_{\ell }^{2}$, as a function of Na_2_SO_4_ concentration. While the variance of the cell length increases monotonically up to 0.8 m, the mean cell length appears to change non-monotonically; it decreases for 0.2 m and 0.4 m, increases for 0.6 m and 0.8 m, and decreases again at 1.0 m Na_2_SO_4_. Cell length exhibits large heterogeneities in morphology at 0.6 m and 0.8 m sodium sulfate concentrations as indicated by the coefficient of variation (CV). While the elongated cells are responsible for an increase in the average cell length, both shortened and elongated cells contribute to the variance of the cell length. Shortened cells upon increasing salt concentration may arise due to water efflux caused by osmotic pressure difference. Increased fraction of elongated cells at high salt concentration suggests that while the cells still grow, the propensity of cell division decreases with increasing salt concentration. The pH of the media decreases with increasing salt concentration with pH being 6.45±0.1 at the highest concentration of Na_2_SO_4_. To test whether the pH decrease could be responsible for the changes in the growth and morphology of the cells, we performed experiments by changing the pH of the media to 6.45±0.1, the pH corresponding to the highest salt concentration (1 m) where the cell growth is inhibited. For these experiments, the pH of the media was adjusted by adding an appropriate amount of hydrochloric acid (HCl) to the M9 media with the supplement of 0.4% glucose, 0.4%succinate, and 2 mM MgSO_4_. The results of the growth and morphology of the cells are shown in Figure S1 in Supplementary Materials. More than a thousand cells were used to obtain the statistics of the cell length. We find that decrease of pH results in slight increase of the doubling time ($\tau_{d}=60\pm 5$ min) compared to cells grown in pH =7.0 ($\tau_{d}=45\pm 4$ min). The morphology of the cells exhibits negligible effect as shown in the representative images and the probability distribution of the cell length at pH =6.45 (see Figure S1B,C in the Supplementary Materials).

### 3.3. Reversibility of Cell Morphology upon Removal of the Salt Stress

**Figure 7:**
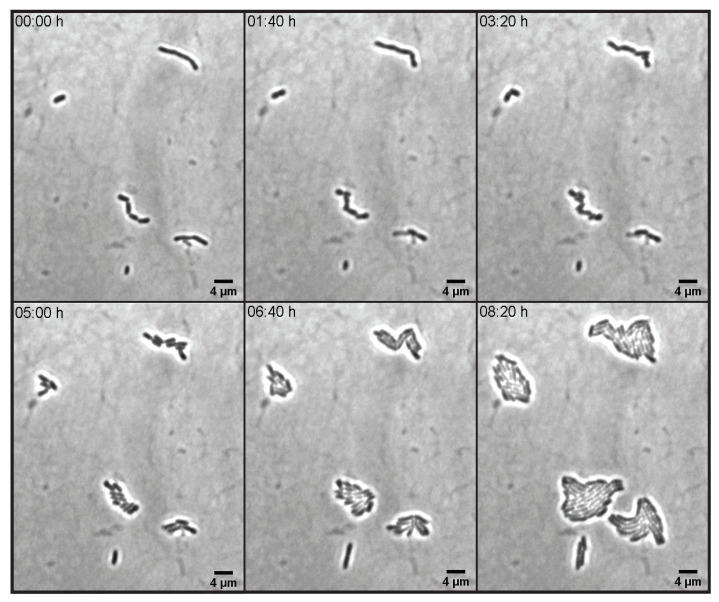
Time-lapse images of cells obtained at 0.8 m Na_2_SO_4_ and subsequently grown on a thin LB-agar chamber without sodium sulfate.

After examining the changes in the morphology of the bacterial cells under different concentrations of sodium sulfate, we then questioned whether the effects of sodium sulfate on *E. coli* are permanent. For this, we look at the changes in the morphology of the cells after the salt stress is removed. The cells were cultured in 0.8 m Na_2_SO_4_ to OD${}_{6}00\approx 0.8$. The sample for imaging was prepared by adding a small sample (≈1 μL) of cells grown at 0.8 m Na_2_SO_4_ to a thin LB-agar chamber (without Na_2_SO_4_). Time-lapse images of cell growth and division were obtained at room temperature (≈23 ${}^{\circ }$C). Figure 7 shows an image sequence of the bacterial cells acquired over 500 min. We find that all the cells were able to go back to the morphology of the control cells after a few generations. A reversibility of the cell morphology movie from the time-lapse images is provided in the Supplementary Materials (Video S1).

### 3.4. Expression of Gene Involved in the Transport of Water, Sodium, Uptake and Metabolism of Sulfate, and Fatty Acid Production

**Figure 8:**
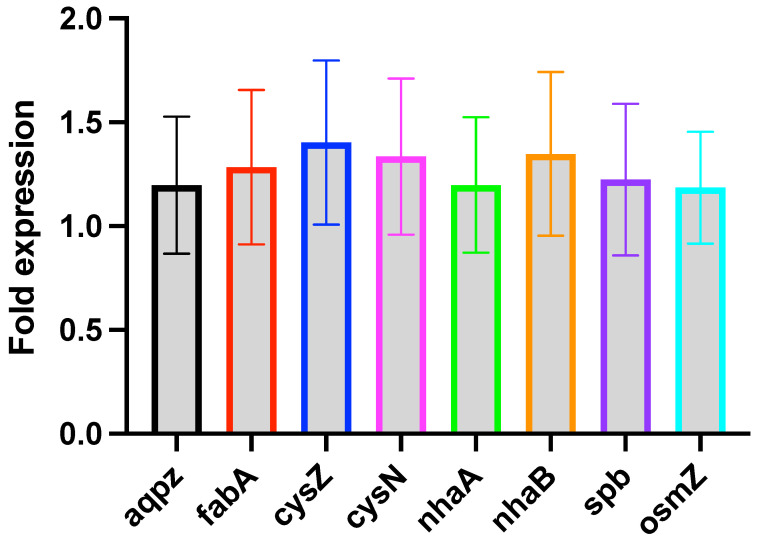
Comparative expression of various genes involved in water transport (*aqpZ*), sodium transport (*nhaA, nhaB*), sulfate transport (*cysZ*, *sbp*), sulfate metabolism (*cysN*), a global transcriptional regulator (*osmZ*), and fatty acid production (*fabA*). We do not find significant changes in the expression of these genes ($\langle \Delta \Delta C_{t}\rangle \leq 0.5)$ at high salt concentration during the exponential growth phase. *cysZ* and *nhaB* exhibit slight upregulation within the error bar. However, this is not conclusive from our data due to large error bar with the $\langle \Delta \Delta C_{t}\rangle \leq 0.5$.

Gene regulation is fundamental to cellular functions in response to environmental changes. We specifically looked at the expression of genes involved in osmotically driven transport of water (*aqpZ*), sodium transport (*nhaA*, *nhaB*), sulfate transport (*cysZ*, *sbp*), sulfate metabolism (*cysN*), fatty acid production (*fabA*), and a global transcriptional regulator (*osmZ*). We compared the expression of these genes between the bacteria grown in 0 m Na_2_SO_4_ (control cells) and 0.8 m Na_2_SO_4_ (sample cells) during the mid-exponential growth phase. Figure 8 shows the fold expression changes of these genes, $2^{-\Delta \Delta Ct}$, between the control and the sample cells. The data are obtained by averaging over two biological and two technical replicates. The error bars were estimated from the standard error of the data for ΔCt. A value of ΔΔCt>0 (fold expression greater than one) would mean upregulation of the gene under the salt stress, while ΔΔCt<0 (fold expression less than one) would mean downregulation. The gene expression differences between the control and the sample cells are insignificant for all the genes ($\langle \Delta \Delta Ct\rangle \leq 0.5$). The expressions of *cysZ* and *nhaB* show slight upregulation. However, this is inconclusive from our data due to large variability with $\langle \Delta \Delta Ct\rangle <0.5$. Earlier studies of *aqpZ* expression in media containing NaCl found that it is downregulated in the exponential growth phase [55]; however, later studies of the effect of NaCl [56], and MgSO_4_ [48] did not find any changes in the expression of *aqpZ* in the exponential growth phase. Since we conducted our experiments for the gene expression in the exponential growth phase, our result of *aqpZ* expression at high salt concentration is consistent with Soupene et al. [56] and Nepal et al. [48]. The expressions of sodium/proton antiporters (*nhaA, nhaB*) shows an insignificant fluctuation, suggesting that the intracellular levels of sodium does not change between the control and the sample cells or the change is negligible. This is also consistent with the fact that the expression of *nhaA* is affected by a global regulator H-NS [57], that is transcribed by *osmZ*, that shows minimal changes in our results. Moreover, neither the sulfate transport nor the sulfate metabolism seem to be affected significantly at high concentration of sodium sulfate during the exponential growth phase.

### 3.5. Osmotic Pressure as the Limiting Factor for the Growth Inhibition

A comparison between the cell viability (fraction of live cells in the population in the exponential growth phase) in magnesium sulfate [48] and sodium sulfate suggests that bacterial cells are more viable in MgSO_4_ as compared to Na_2_SO_4_ at a given salt concentration. At a given salt concentration, the ionic strength of the aqueous solution in MgSO_4_ is larger as compared to Na_2_SO_4_. This suggests that ionic strength is not a limiting factor. Water activity and osmotic pressure of the growth medium also affect the growth and viability of cells [58,59,60,61]. Experimental data of osmotic coefficient and water activity are available for different aqueous solutions of various salts [62] at temperature 25 ${}^{\circ }$C, but not at 37 ${}^{\circ }$C. Since our experiments were performed at 37 ${}^{\circ }$C, we calculated the osmotic coefficient and osmotic pressure using Pitzer model [63]. The Pitzer model has been used successfully to describe the equilibrium solubility and composition for a large number of electrolytes to very high concentrations [64,65]. According to the Pitzer model, the osmotic coefficient, ϕ, of an aqueous solution of salt is calculated from

(3) $$\phi -1=\frac{2}{\sum m_{i}}\left(-\frac{A^{\phi }}{1+bI^{1/2}}+\sum \sum m_{c}m_{a}\left(B_{ca}^{\phi }+ZC_{ca}\right)\right),$$

where $m_{c}$ and $m_{a}$ are the molalities of the cations and the anions, respectively. *I* is the ionic strength of the solution, defined as

$$I=0.5\sum_{i}z_{i}^{2}m_{i}$$

where $z_{i}$ is the charge on the ith species. $A^{\phi }$, $B_{ca}^{\phi }$, and $C_{ca}$ are the model parameters, *b* is a constant, and *Z* is defined as:

$$Z=\sum m_{i}\left|z_{i}\right|.$$

For a detailed discussion of the Pitzer model, the readers should refer to Refs. [63,64,65,66] and the references therein. The parameters and their temperature dependence for Na_2_SO_4_ are taken from Moller et al. [67], and parameters for MgSO_4_ are taken from Marion et al. [66]. The water activity, $a_{W}$, is given by:

(4) $$a_{W}=\exp\left(-\frac{\phi \sum m_{i}}{55.50844}\right).$$

The osmotic pressure, Π, is related to the osmotic coefficient and the number of moles of cation and anion by

(5) $$\Pi =RT\frac{M_{s}}{{\bar{V}}_{s}}\nu m\phi ,$$

where *R* is the Universal Gas constant, *T* is the temperature, $M_{s}/{\bar{V}}_{s}$ is the mass of the solvent per unit volume, *m* is the molality of the solution, and $\nu =\nu_{c}+\nu_{a}$ is the total number of moles of cations and anions per mole of the salt in the solution. For example, the total number of moles, ν, for Na_2_SO_4_ and MgSO_4_ are 3 and 2, respectively. We have assumed that ${\bar{V}}_{s}$ is of the pure solvent.

**Figure 9:**
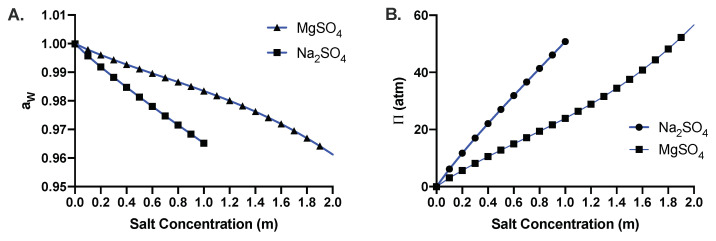
(**A**) Water activity, $a_{W}$, and (**B**) osmotic pressure, Π, as a function of concentration of aqueous solutions of sodium and magnesium sulfate at *T* = 37 ${}^{\circ }$C, computed using the Pitzer model.

Figure 9A,B shows the calculated values of the water activity and the osmotic pressure as a function of salt concentration, respectively. Figure 9A shows that, even though the water activity decreases with increasing salt concentrations for sodium sulfate and magnesium sulfate, the value is still larger than the limiting water activity $a_{W}\approx 0.6$ [58], where the microbial growth is inhibited. However, limiting water activity can vary depending on the organisms [60,61]. The concept of water activity is well established [68], and it has been used as an effective determinant for the physiological state of cells [58,61,69]. Even though water activity is a useful index, it does not provide ample information about failure of a particular cellular process as it may affect many cellular processes [70,71]. Moreover, water activity does not provide a complete determination of the state of water, particularly in protein unfolding [72]. Water activity is also related to osmotic pressure. As a result of this relationship, limiting water activity also entails the corresponding limiting osmotic pressure (see Figure S2 in the Supplementary Materials). Our gene expression results show an insignificant increase in the uptake of sodium and sulfate ions at 0.8 m Na_2_SO_4_. Our results further suggest that there is a gradual increase in membrane deterioration with increasing salt concentration as evident from the propidium iodide staining. The resulting cell death may arise from membrane rupture due to osmotic pressure difference between intracellular and extracellular regions. We next explore the limit of growth in terms of osmotic pressure as it provides a mechanistic picture of growth inhibition in our context.

**Figure 10:**
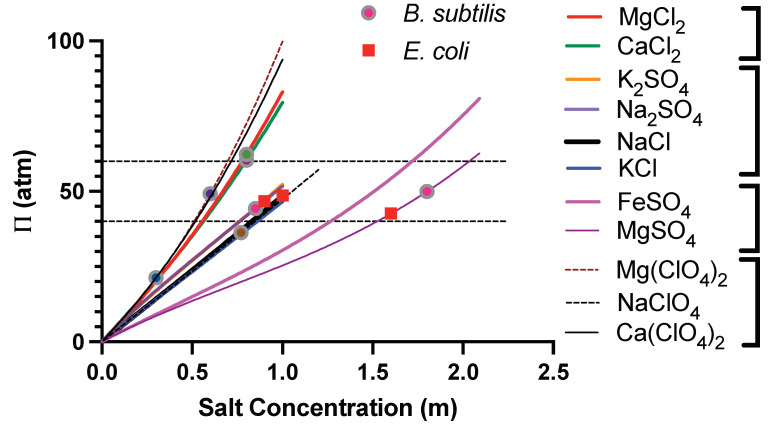
Osmotic pressure, Π, as a function of concentration of aqueous solutions of various salts at *T* = 37 ${}^{\circ }$C, computed using the Pitzer model. We also show the osmotic pressures corresponding to the limiting salt concentrations of growth of *E. coli* and *B. subtilis*.

For both Na_2_SO_4_ and MgSO_4_ solutions, the changes in the external osmotic pressure of the growth media could be as high as 50 atm (not counting the osmotic pressure due to M9 media) for the range of salt concentrations considered here. Furthermore, the osmotic pressure of Na_2_SO_4_ solution increases more rapidly as compared to MgSO_4_ solution with increasing salt concentration. To investigate whether the changes in the osmotic pressure of the external media is the limiting factor for growth of the bacterial cells, we then compared the osmotic pressures where the cell growth is inhibited in both MgSO_4_ and Na_2_SO_4_, henceforth termed the limiting osmotic pressure. Cell growth ceases at 1.66 m MgSO_4_ [48] and 1.0 m Na_2_SO_4_. The osmotic pressures corresponding to these concentrations are ≈44 atm and ≈50 atm, respectively, which are similar in magnitude. We further use the recent data of Stevens et al. [54] that have performed a systematic study of the limiting salt concentrations for the growth of *B. subtilis*, another non-halophilic bacterium, under various salt compositions. We calculated the osmotic pressure of nine different salts—(i) MgCl_2_, (ii) CaCl_2_, (iii) K_2_SO_4_, (iv) NaCl, (v) KCl, (vi) FeSO_4_, (vii) Mg(ClO_4_)_2_, (viii) NaClO_4_, and (ix) Ca(ClO_4_)_2_, besides Na_2_SO_4_ and MgSO_4_ as a function of their aqueous concentrations using the Pitzer model at *T* = 37 ${}^{\circ }$C. The parameters for the Pitzer model (Equation (3)) and their temperature dependence is taken from Ref. [66]. In Figure 10, we show the osmotic pressure, Π, as a function of concentration of various salts. We also show the limiting concentration of salts for growth inhibition found for *B. Subtilis* by Stevens et al. (as circles) and the limiting salt concentrations from our studies of *E. coli* in MgSO_4_ and Na_2_SO_4_ (as squares). We find that besides one exception Ca(ClO_4_)_2_, the osmotic pressure corresponding to the limiting salt concentration for different salts lies between 40 atm and 60 atm, irrespective of the nature of the salt. Enhanced chaotropicity of Ca(ClO_4_)_2_ could be a plausible factor for the discrepancy between Ca(ClO_4_)_2_ and other salts [61]. It is also worthwhile to note that the limiting osmotic pressure is the same for a gram-positive bacterium (*B. subtilis*) and a gram-negative bacterium (*E. coli*).

## 4. Discussion

In summary, we have studied the effect of high concentrations of sodium sulfate on cell growth, death, morphology, cell division, and gene expressions of a halotolerant bacterium *E. coli*. We find that *E. coli* can tolerate moderately high concentrations of sodium sulfate. The growth rate decreases with increasing salt concentration up to 0.8 m Na_2_SO_4_, and ultimately ceases beyond ≥1.0 m. Our results are similar to earlier studies on the effect of on the growth of *E. coli* under high concentration of magnesium sulfate [48]. We find that cell death increases with increasing salt concentrations.

We also examined the cell division and cell morphology under high concentrations of sodium sulfate. We find that average cell length decreases up to 0.4 m and increases between 0.4 m and 0.8 m Na_2_SO_4_. For 0.6 m and 0.8 m sodium sulfate concentrations, cells exhibit three distinct morphologies—cells shorter than the control cells, cells comparable to control cells, and elongated cells. The shortening of cells with increasing Na_2_SO_4_ concentration may arise due to water efflux, while elongated cells in the population arise due to lack of cell division. Cell elongation has also been reported for other stressors such as high hydrostatic pressure [50,73], high concentration of magnesium sulfate [48], high temperature [74], antibiotic treatment [75], and DNA damage [76]. There are two important questions that remain unanswered—(i) why the cell elongation occurs, and whether it is some form of evolutionary adaptation of the cells under stress conditions? and (ii) what are the physical mechanisms responsible for the elongation/filamentation of cells? While the former is an important inquiry, it is also challenging to answer. Therefore, we will only discuss the plausible physical mechanisms for cell filamentation observed here. Cell division in *E. coli* is coordinated by a complex divisome machinery involving a large number of proteins, including FtsZ, FtsA, FtsI, FtsL, FtsN, FtsQ, FtsW, ZipA, ZapA, MinD, and MinE, among others [77,78]. FtsZ, in particular, is involved in the synthesis of the division septum, and is essential for the constriction of the septal wall [79,80]. Lack of FtsZ in *E. coli* leads to cell filamentation [81,82]. The presence of FtsZ itself does not guarantee the cell division, but the polymerization of FtsZ into Z-ring at the site of constriction is critical for cell division [83,84]. In the cases of high hydrostatic pressure, it was shown that the propensity of FtsZ localization and polymerization into Z-ring decreases, and hence the inhibition of cell division at high pressure [85]. Many of the cell division proteins require anchoring to the membrane in order to be localized [77]. Therefore, for the cell division machinery to function correctly, a large number of biophysical processes must be considered, including membrane integrity, localization, and polymerization kinetics of cell division proteins. Moreover, the dynamics of cell division is coupled to the cell wall elongation and cell growth [86] that also requires localization and polymerization of many cytoskeletal proteins including MreB [87,88]. Our results of cell viability suggest cell wall damage and deterioration of membrane integrity at high salt concentration may affect localization and polymerization kinetics of many of the proteins involved in cell division and cell growth. Further experiments would be required to answer some of these questions. The heterogeneity in cell division, and hence morphology may arise due to heterogeneities in gene expression and stochastic nature of the cellular processes [50,89,90]. We find that the cells grown at high salt concentrations revert to normal morphology upon removing the salt stress. The dynamics of the reversibility of the cell division with heterogeneous morphology upon the removal of the salt stress will be a subject of future investigation.

We also investigated the expression of genes involved in the regulation of various cellular processes. Specifically, we compared the expression of different genes between the control cells (0 m Na_2_SO_4_) and the cells grown at high salt concentration (0.8 m Na_2_SO_4_). Our results indicate that the expression level of these genes does not change significantly in the exponential growth phase at 0.8 m Na_2_SO_4_. AqpZ, an aquaporin channel, is implicated in the osmotically driven transport of water [55,56,91]. Earlier studies of hyperosmotic conditions on *E. coli* indicate that *aqpZ* is upregulated in the stationary growth phase and not in the exponential growth phase [56], which is consistent with no upregulation for *aqpZ* in the exponential growth phase. The shrinkage of cells observed at high salt concentrations could arise due to slow diffusion of water between the cell and the external environment. CysZ, a high affinity sulfate transporter mediates the transport of sulfate for cysteine synthesis [92], while Sbp, a periplasmic protein, is involved in the uptake of sulfate and thiosulfate [93]. Furthermore, the expression of both *cysZ* and *sbp* are not affected at high sodium sulfate concentration, indicating no up/down regulation of sulfate transport by CysZ or Sbp. If there is an increase in sulfate uptake with increasing Na_2_SO_4_ concentration, it is likely carried out by other transporters such as CysP, as found for MgSO_4_ [48]. The production of CysN, a unit of sulfate adenylyltransferase required for sulfate assimilation [94], does not change at high Na_2_SO_4_ concentration. Furthermore, we find that the activity of sodium antiporters, *nhaA* and *nhaB*, show no changes in the presence of high concentration of Na_2_SO_4_. In *E. coli*, *nhaA* expression is induced by the increased intracellular levels of Na${}^{+}$ [95]. No expression changes of *nhaA* suggests that either the intracellular level of Na${}^{+}$ does not change or is regulated by another mechanism. H-NS, transcribed by *osmC*, regulates the expression of *nhaA* [96]. Therefore, our results of no expression changes of *nhaA* is consistent with no expression changes of *osmC*. Fatty acids are the precursor to phospholipids, the building blocks of cell membrane. By changing the degree of saturation, cells can regulate the rigidity/fluidity of cell membrane. Membrane fluidity decreases under hyperosmotic conditions [97,98]. Therefore, one may expect that in the presence of high external osmotic pressure, cells may rigidify the membrane by increasing the levels of saturated fatty acids and/or decreasing the proportion of unsaturated fatty acids. Overproduction of *fabA* in *E. coli* is shown to increase the levels of saturated fatty acids with no increase of unsaturated fatty acid levels [99]. We find that the expression of *fabA* does not change at high concentration (0.8 m) of sodium sulfate. An earlier study on the effect of sodium chloride on *E. coli* suggests a monotonous increase of *fabA* expression with the salt concentration up to 5% NaCl (0.85 m) [100], in the presence of glycine betaine. The presence of glycine betaine, an osmoprotectant, in the study of Ref. [100] makes it difficult to compare with our results.

Several factors affect the growth of a cell and its inhibition in hyperosmolar environments including chaotropic and kosmotropic nature of ions, ionic strength, water activity or osmotic pressure. We use our data of *E. coli* in Na_2_SO_4_ and MgSO_4_ [48] along with the limiting salt concentration data of Stevens et al. [54] for *B. subtilis* in different salts, and find that the osmotic pressure difference may play a major role in determining the growth inhibition of non-halophilic bacteria at high salt concentrations. Specifically, limiting osmotic pressures for all the salts lie between 40 atm and 60 atm except Ca(ClO_4_)_2_, for which the limiting osmotic pressure is smaller (≈25 atm). The reason for cell growth inhibition in Ca(ClO_4_)_2_ at a smaller osmotic pressure (or larger water activity) may stem from enhanced chaotropicity of Ca(ClO_4_)_2_ [61].

Saline environments are also proposed for other icy bodies, such as Ceres [101,102], and therefore our results are important for understanding the potential habitability of these moons. The conditions on icy moons, including Europa, do not only include hyperosmolar environments but also high pressure and presumably extremes of temperature. We have only investigated the behavior of *E. coli* under one condition, varying concentrations of sodium sulfate. Future studies on the behavior of cells under a combination of environmental conditions, such as pressure, temperature, and high salt concentration or a combination of salts, are needed to further understand the viability of a non-halophilic bacterium under the physicochemical conditions that may exist on these celestial bodies.

## Figures and Tables

**Table 1:** Dependence of pH of the media on Na_2_SO_4_ concentration.

Na_2_SO_4_ (m)	pH (±0.1)
0.0	7.00
0.2	6.82
0.4	6.77
0.6	6.62
0.8	6.55
1.0	6.45

**Table 2:** List of the primers used.

Gene	Primer	Sequence (5${}^{\prime }$-3${}^{\prime }$)
16s *rRNA*	Forward Reverse	TCGTCAGCTCGTGTTGTGAA AGGGCCATGATGACTTGACG
*aqpZ*	Forward Reverse	AGCATTCACCAGGCGGTTAT TCAGGGTTAAGGCCAGACCA
*cysN*	Forward Reverse	ATCGCCACGACCAGATGTTT CGAGCAGTACACCCGCAATA
*cysZ*	Forward Reverse	CGTTCCGGACTGGCTACAAT CGTGCTTCCAGTTGTTCAGC
*fabA*	Forward Reverse	AATTTCACTTCGCCAACGCC TCTGTGGTTCTTCGGATGCC
*nhaA*	Forward Reverse	TGAAAGAGAAGCATGGGCGT GCAGAATGGAGGTCAAGCCA
*nhaB*	Forward Reverse	TCTTGCAGGTCGGTGTCTTC GTCGCTCTCTTTTTGCGTGG
*osmZ*	Forward Reverse	TTCGTTCGGGTCAATACCGT ACGCTGGAAGAAATGCTGGA
*sbp*	Forward Reverse	CACGCCGAGTGAGTCTATCC CGGTAGTAGTTTTTCGCGGC

**Table 3:** Cell length statistics.

Na_2_SO_4_ (m)	$\langle \ell \rangle$ (μm)	$\sigma_{\ell }^{2}$ (μm${}^{2}$)	CV (%)
0	2.04	0.22	22.77
0.2	1.64	0.26	31.34
0.4	1.93	0.41	31.81
0.6	2.69	2.54	59.29
0.8	3.78	4.74	57.57
1.0	1.66	0.28	32.02

## Data Availability

Data will be made available upon publication.

## Supplementary Materials

The following are available online at https://www.mdpi.com/article/10.3390/microorganisms10020274/s1, Figure S1: Cell growth and cell morphology at pH 6.45, Figure S2: Limiting water activity of *B. subtilis* and *E. coli* in different salts, Video S1: Reversibility of morphology of cells upon removal of the salt stress.
