# Supplementary material for: Morphological Phenotypes, Cell Division, and Gene Expression of Escherichia coli under High Concentration of Sodium Sulfate
Source: Microorganisms. 2022 Jan 25;10(2):274. doi: 10.3390/microorganisms10020274 (PMC8875244; doi:10.3390/microorganisms10020274)
Supplement: Supplementary file 1 [file microorganisms-10-00274-s001.zip › supplementary/supplementary_nkMDPI.pdf]

Article

Supplementary Information: Morphological phenotypes, cell division, and gene expression of *Escherichia coli* under high concentration of Sodium Sulfate

Khanh Nguyen[1] and Pradeep Kumar [1]*

[1] Department of Physics, University of Arkansas, Fayetteville, AR, 72701 USA

Version January 13, 2022 submitted to Microorganisms

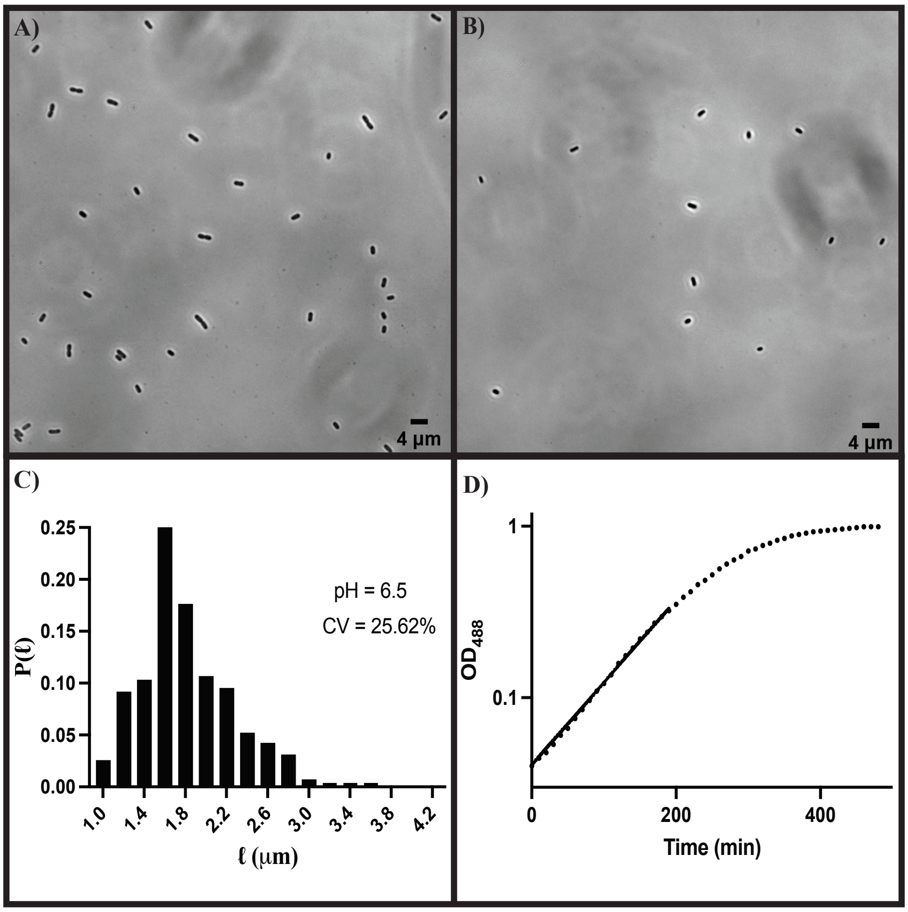

Fig. S 1. Representative images of cells grown at (A) at pH= 7.0, and (B) at pH= 6.45. (C) Probability distribution of cell length at pH=6.45. (D) Linear-log plot of the growth curve of the cells at pH= 6.45. The doubling time ($\tau_d = 60 \pm 5$ min) decreases by a small amount compared to cells grown in pH= 7. Both the cell morphology and cell growth exhibit negligible changes in morphology compared to cells grown in 1.0 m Na_2SO_4, corresponding to the lowest pH= 6.45.

© 2022 by the authors. Submitted to *Microorganisms* for possible open access publication under the terms and conditions of the Creative Commons Attribution (CC BY) license (http://creativecommons.org/licenses/by/4.0/).

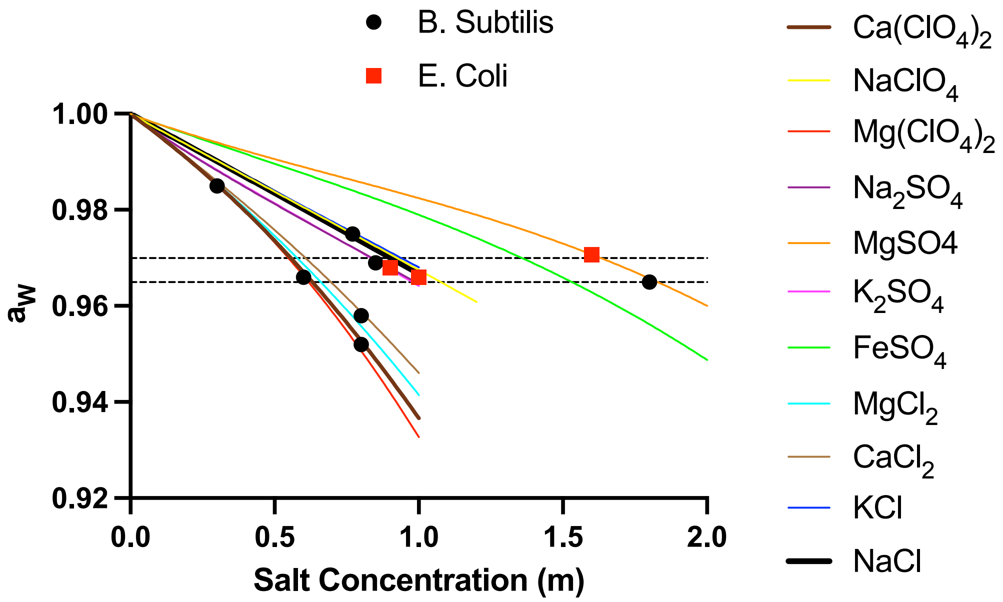

Fig. S 2. Water activity, a_w, as a function of salt concentration of various salts. We also show the limiting water activity for *E. coli* (squares) and *B. Subtilus* (circles) in different salts. Dotted lines are constant a_w lines for $a_w = 0.965$ and $a_w = 0.970$.
